# Complement component 7 (C7), a potential tumor suppressor, is correlated with tumor progression and prognosis

**DOI:** 10.18632/oncotarget.13294

**Published:** 2016-11-11

**Authors:** Lisha Ying, Fanrong Zhang, Xiaodan Pan, Kaiyan Chen, Nan Zhang, Jiaoyue Jin, Junzhou Wu, Jianguo Feng, Herbert Yu, Hongchuan Jin, Dan Su

**Affiliations:** ^1^ Laboratory of Cancer Biology, Provincial Key Lab of Biotherapy in Zhejiang, Sir Runrun Shaw Hospital, Medical School of Zhejiang University, Hangzhou, China; ^2^ Cancer Research Institute, Zhejiang Cancer Hospital & Key Laboratory Diagnosis and Treatment Technology on Thoracic Oncology of Zhejiang Province, Hangzhou, China; ^3^ Tissue Bank of Zhejiang Cancer Hospital, Hangzhou, China; ^4^ Cancer Epidemiology Program, University of Hawaii Cancer Center, Hawaii, USA

**Keywords:** complement component 7, tumor progression, prognosis, ovarian cancer, non-small cell lung cancer

## Abstract

Our previous study found copy number variation of chromosome fragment 5p13.1-13.3 might involve in the progression of ovarian cancer. In the current study, the alteration was validated and complement component 7 (C7), located on 5p13.1, was identified. To further explore the clinical value of C7 in tumors, 156 malignant, 22 borderline, 33 benign and 24 normal ovarian tissues, as well as 173 non-small cell lung cancer (NSCLC) tissues along with corresponding adjacent and normal tissues from the tissue bank of Zhejiang Cancer Hospital were collected. The expression of C7 was analyzed using reverse transcriptase quantitative polymerase chain reaction. As a result, the C7 expression displayed a gradual downward trend in normal, benign, borderline and malignant ovarian tissues, and the decreased expression of C7 was correlative to poor differentiation in patients with ovarian cancer. Interestingly, a similar change of expression of C7 was found in normal, adjacent and malignant tissues in patients with NSCLC, and low expression of C7 was associated with worse grade and advanced clinical stage. Both results from this cohort and the public database indicated that NSCLC patients with low expression of C7 had a worse outcome. Furthermore, multivariate cox regression analysis showed NSCLC patients with low C7 had a 3.09 or 5.65-fold higher risk for relapse or death than those with high C7 respectively, suggesting C7 was an independent prognostic predictor for prognoses of patients with NSCLC. Additionally, overexpression of C7 inhibited colony formation of NSCLC cells, which hints C7 might be a potential tumor suppressor.

## INTRODUCTION

The complement system is a phylogenetically ancient makeup of the innate immune system and plays a vital role in immune surveillance and homeostasis [1, 2]. After activated through a linear cascade of separate pathways known as the classical, lectin and alternative pathways, complements trigger irreversible assembly of a multi-protein pore in cell membranes, the membrane attack complex (MAC) which functions as the cytolytic effector unit of complement system [1–3]. Apart from directly lysing malignant transformed cells, the complements cascade also enhances antibody-dependent cell-mediated cytotoxicity (ADCC) against tumor cells [2, 4].

The complement component 7 (C7) is a terminal component of the complement cascade and its insertion into the lipid bilayers is a critical limiting factor for the formation of MAC [5–7]. It is generally recognized the inherited deficiency of C7 is correlated with an increased susceptibility to a wide range of Gram-negative bacteria [8, 9]. Although the majority of systemic C7 is derived from hepatic and marrow sources [10], an estimated thirty percent of C7 from local synthesis has been described also important in the formation of MAC [5, 6, 10]. However, the role of C7 synthesized in tumors was few studied.

In the current study, C7 was identified as a potential tumor suppressor in ovarian cancer and non-small cell lung cancer (NSCLC). Moreover, it is an independent predictor for patient outcome.

## RESULTS

### Comparison of chromosome fragment 5p13.1-13.3 between cell lines

**Figure 1:**
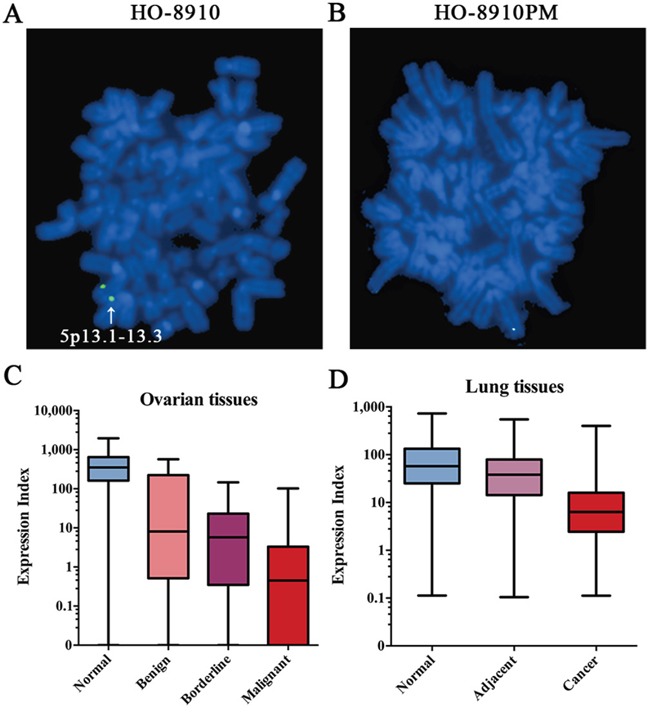
The deletion of C7 in tumor progression **A & B**. Detection of chromosome fragment 5p13.1-13.3 by FISH in HO-8910 and HO-8910PM. Green dots located on the sister chromatid were determined as real signals. Original magnification ×400. **C**. Different C7 expression levels in ovarian tissues with diverse biological behaviors. The C7 mRNA expression of 4.2% (1/24) normal tissues, 15.2% (5/33) benign tissues, 13.6% (3/22) borderline tissues and 26.9% (42/156) malignant tissues was too low to detect and thereby the C7 expression of those tissues was considered negative (Expression Index = 0). Analyzed by the Kruskal-Wallis and the Dunn test, the C7 expression values were found significantly different between malignant and any other group. **D**. Different C7 expression levels in lung tissues with diverse biological behaviors. Significant difference of C7 expression was found between any two of lung groups.

Our previous study [11] found that the high metastatic ovarian cancer cell line HO-8910PM had less copy number in chromosome fragment 5p13.1-13.3 than its parental cell line HO-8910. In this study, the fluorescent *in situ* hybridization (FISH) assay was carried out to verify this observation. By using specific biotin-labeled BAC probes which mapped into 5p13.1-13.3, one copy chromosomal hybridization signal was found in HO-8910 (Figure 1A), while no signal was detected in HO-8910PM (Figure 1B), suggesting loss of heterozygosity of chromosome fragment 5p13.1-13.3 in HO-8910 and homozygous deletion of chromosome fragment 5p13.1-13.3 in the parental cell line, which was identical with our previous CGH results. Combining with our previous microarray data on gene expression profiles [12] which showed that the C7 had the most significant decrease of expression among genes located on chromosome fragment 5p13.1-13.3 in HO-8910PM compared with HO-8910, the C7 was further studied.

### The clinical and prognostic values of C7 in ovarian cancer patients

Firstly, the expression of C7 in ovarian tissues with diverse biological behaviors was analyzed. As a result, the mRNA expression levels of C7 in normal, benign, borderline and malignant ovarian tissues showed a gradual declining trend (Figure 1C). The median Expression Indexes of C7 expression were 351.13, 8.09, 5.77 and 0.45 in normal, benign, borderline and malignant ovarian tissues and significant difference of C7 expression was found between malignant and any other group.

**Figure 2:**
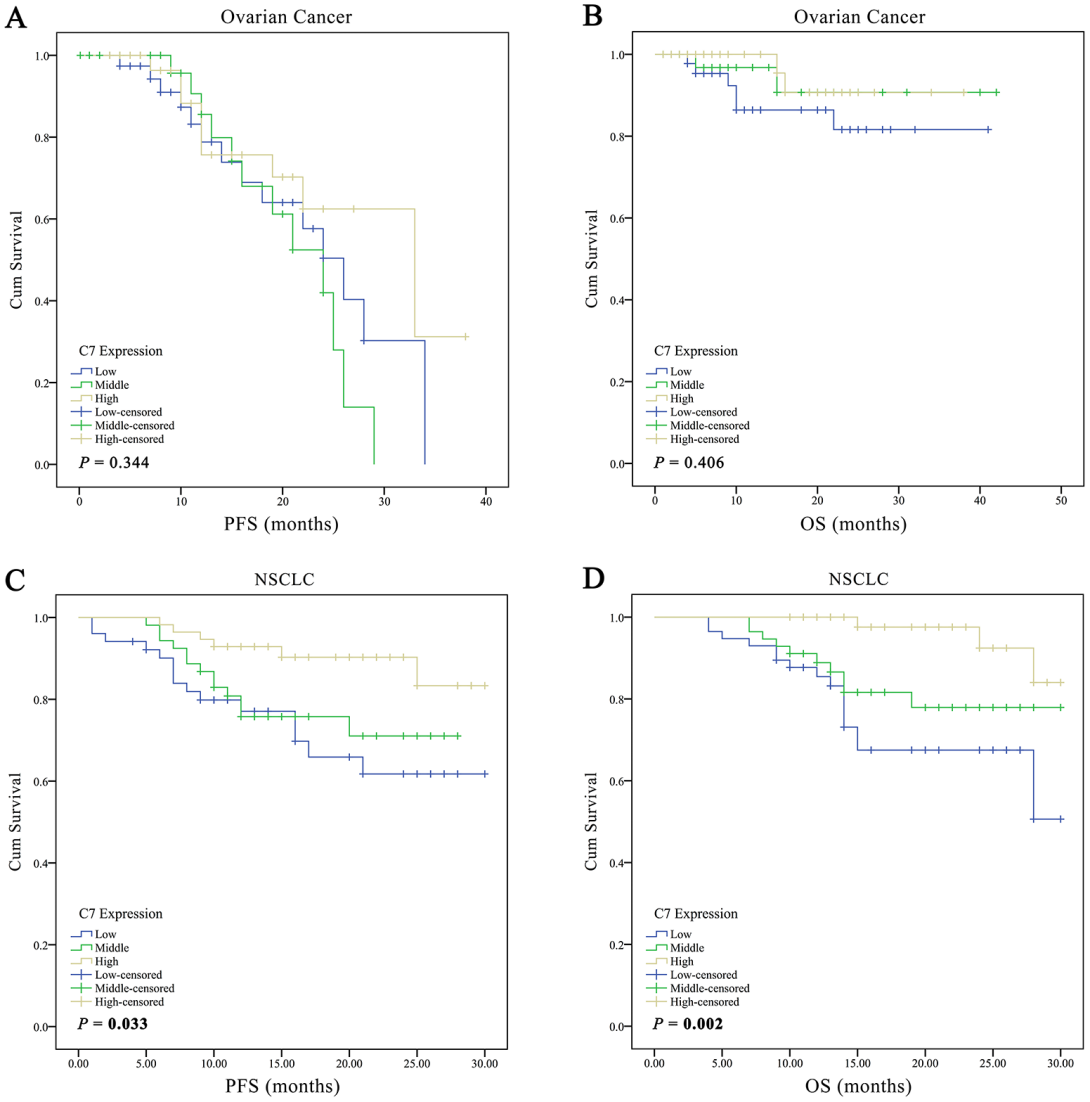
Kaplan-Meier curves according to the C7 expression levels for progression free survival and overall survival of patients with ovarian cancer or non-small cell lung cancer The survival curves were developed using Kaplan-Meier estimator and the *P* value was calculated through Log-rank test. *PFS* progression free survival, *OS* overall survival, *NSCLC* non-small cell lung cancer.

**Figure 3:**
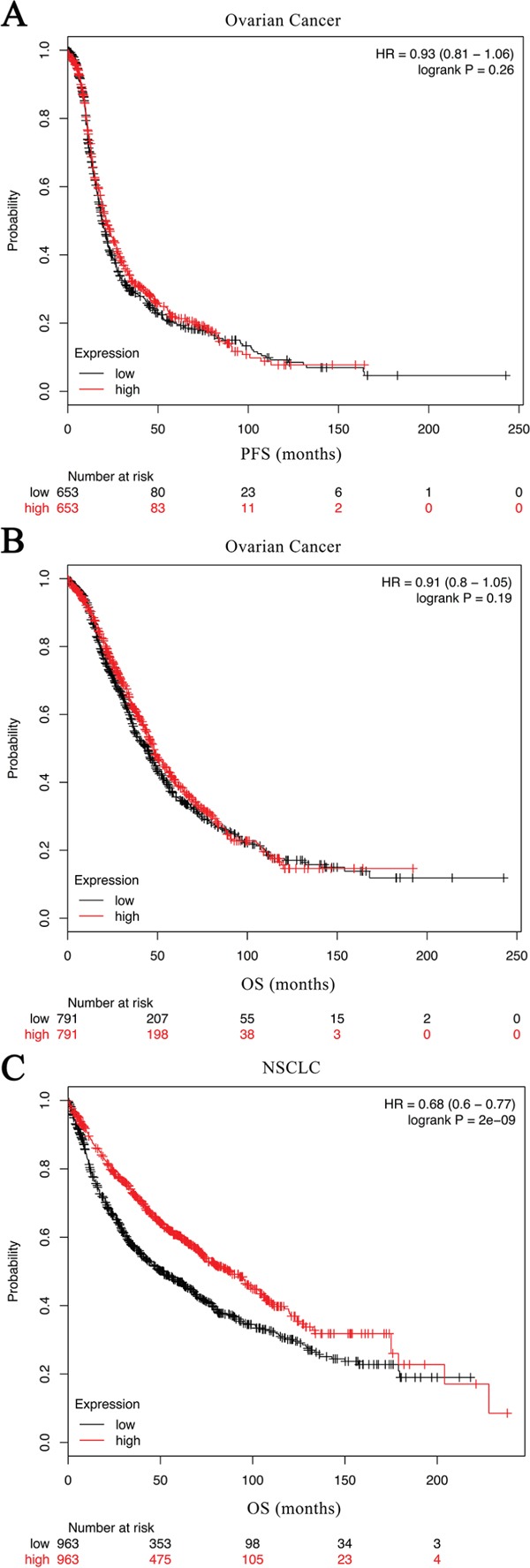
Kaplan-Meier curves created by the public database and web application KM plotter (http://kmplot.com/analysis/) based on the C7 expression levels Analysis for PFS of NSCLC was unavailable in this database. *HR* hazard ratio, *PFS* progression free survival, *OS* overall survival, *NSCLC* non-small cell lung cancer.

Subsequently, the relation between C7 expression and clinicopathological factors, as well as its relation with outcomes in 156 ovarian cancer patients was investigated. As displayed in Table 1, both Mann-Whitney U test and chi-square test found that the expression of C7 was significantly related to tumor grade in patients with ovarian cancer. Patients with inferior tumor grade had a low expression level of C7. Nonetheless, the Cox regression model and Kaplan-Meier analysis didn't find any significant association between C7 expression and prognosis in patients with ovarian cancer (Table 2, Figures 2A and 2B). The public database showed similar results (Figures 3A and 3B, http://kmplot.com/analysis/index.php?p=service&cancer=ovar) [13].

**Table 1:** Association of C7 mRNA expression with clinicopathological factors in patients with ovarian cancer

Factors	Case, n (%)	C7 expression	*P* value	C7 expression			*P* value
		Median (5th-95th)		Low, n (%)	Middle, n (%)	High, n (%)	
**Age (year)**			0.447				0.199
<60	110 (70.5)	0.33 (<0.01-21.29)		37 (69.8)	41 (78.8)	32 (62.7)	
≥60	46 (29.5)	0.84 (<0.01-34.18)		16 (30.2)	11 (21.2)	19 (37.3)	
**Histology**			0.630				0.640
Non-serous	42 (26.9)	0.79 (<0.01-22.74)		12 (22.6)	16 (30.8)	14 (27.5)	
Serous	114 (73.1)	0.36 (<0.01-28.17)		41 (77.4)	36 (69.2)	37 (72.5)	
**Grade**			**0.011**				**0.002**
1-2	21 (13.5)	3.15 (<0.01-71.95)		4 (7.8)	3 (6.5)	14 (29.2)	
3	124 (79.5)	0.29 (<0.01-15.76)		47 (92.2)	43 (93.5)	34 (70.8)	
Missing	11 (7.1)						
**Clinical stage**			0.203				0.062
I-II	28 (17.9)	1.57 (<0.01-30.51)		7 (13.2)	7 (13.5)	14 (27.5)	
III-IV	128 (82.1)	0.39 (<0.01-25.49)		46 (86.8)	45 (86.5)	37 (72.5)	
**Residual Tumor**			0.165				0.077
No residual	78 (50.0)	0.67 (<0.01-20.62)		32 (61.5)	20 (39.2)	26 (51.0)	
Residual	76 (48.7)	0.73 (<0.01-27.44)		20 (38.5)	31 (60.8)	25 (49.0)	
Missing	2 (1.3)						

Bold values were statistically significant (*P* ≤ 0.05).

**Table 2:** Correlations between C7 mRNA expression and patient survival estimated by univariate and multivariate Cox regression analyses

	C7 expression	Crude HR	95% CI	*P* value	Adjust HR	95% CI	*P* value
**OC PFS**							
	High	1		0.287	1		0.871
	Middle	1.08	0.62-1.88	0.782	1.05	0.58-1.90	0.869
	Low	0.71	0.40-1.27	0.249	0.89	0.48-1.68	0.727
**OC OS**							
	High	1		0.326	1		0.656
	Middle	1.17	0.64-2.13	0.618	1.36	0.70-2.64	0.368
	Low	0.74	0.40-1.36	0.329	1.10	0.56-2.17	0.782
**NSCLC PFS**							
	High	1		**0.047**	1		0.087
	Middle	2.60	0.99-6.86	0.053	1.68	0.59-4.80	0.333
	Low	3.28	1.27-8.46	**0.014**	3.09	1.09-8.71	**0.033**
**NSCLC OS**							
	High	1		**0.008**	1		**0.025**
	Middle	4.22	1.16-15.40	**0.029**	2.56	0.63-10.35	0.188
	Low	6.95	2.02-23.98	**0.002**	5.65	1.49-21.37	**0.011**

Bold values were statistically significant (*P* ≤ 0.05).

*HR* hazard ratio, *CI* confidence interval, *PFS* progression free survival, *OS* overall survival, *OC* ovarian cancer, *NSCLC* non-small cell lung cancer

### The role of C7 in NSCLC

To further investigate the value of C7 in other types of cancer, a NSCLC cohort was studied. As displayed in Figure 1D, consistent downward trend of C7 expression following tumor progression was discovered in lung tissues. The median Expression Indexes of C7 expression were 57.49, 38.08 and 6.33 in paired normal, adjacent and malignant lung tissues and significant difference of C7 expression was found between any two groups.

As a consequence of correlation analyses, in addition to the tumor grade, the clinical stage was also found significantly associated with the C7 expression in patients with NSCLC (Table 3), and less C7 expression meant worse grade and advanced clinical stage. Notably, as presented in Table 2, the univariate Cox regression analysis implied that depressed C7 expression in tumor tissues was related to poor outcomes in NSCLC patients (crude HR 2.60, 95 % CI 0.99-6.86, *P* = 0.053 for middle level and PFS; crude HR 3.28, 95% CI 1.27-8.46, *P* = 0.014 for low level and PFS; crude HR 4.22, 95% CI 1.16-15.40, *P* = 0.029 for middle level and OS; crude HR 6.95, 95% CI 2.02-23.98, *P* = 0.002 for low level and OS). Similar results were obtained from the Kaplan-Meier estimators (Figures 2C and 2D; *P* = 0.033 for PFS; and *P* = 0.002 for OS) and the public database (Figure 3C, http://kmplot.com/analysis/index.php?p=service&cancer=lung) [14]. What's more, the C7 mRNA expression in NSCLC tissues was identified to be an independent prognostic factor for both disease progression and death after adjusted by gender, age, smoking status, histology type, tumor grade and clinical stage. NSCLC patients with low- and middle-expression C7 would have worse PFS and OS contrasted to those with high-expression C7 (Table 2; adjust HR 1.68, 95% CI 0.59-4.80, *P* = 0.333 for middle level and DFS; adjust HR 3.09, 95% CI 1.09-8.71, *P* = 0.033 for low level and DFS; adjust HR 2.56, 95 % CI 0.63-10.35, *P* = 0.188 for middle level and OS; adjust HR 5.65, 95 % CI 1.49-21.37, *P* = 0.011 for low level and OS).

**Table 3:** Association of C7 mRNA expression with clinicopathological factors in patients with non-small cell lung cancer

Factors	Case, n (%)	C7 expression	*P* value	C7 expression			*P* value
		Median (5th-95th)		Low, n (%)	Middle, n (%)	High, n (%)	
**Gender**			0.653				0.954
Male	141 (81.5)	5.82 (0.54-166.46)		46 (80.7)	48 (82.8)	47 (81.0)	
Female	32(18.5)	7.32 (0.17-145.71)		11 (19.3)	10 (17.2)	11 (19.0)	
**Age (year)**			0.544				0.825
<65	124 (71.7)	5.85 (0.33-106.07)		41 (71.9)	40 (69.0)	43 (74.1)	
≥65	49 (28.3)	7.53 (0.90-264.45)		16 (28.1)	18 (31.0)	15 (25.9)	
**Smoking**			0.714				0.724
Never	36 (20.8)	6.29 (0.38-97.00)		14 (26.4)	11 (20.8)	11 (20.8)	
Ever/current	123 (71.1)	5.83 (0.37-214.44)		39 (73.6)	42 (79.2)	42 (79.2)	
Missing	14 (8.1)						
**Histology**			0.617				0.605
SCC	96 (55.5)	5.55 (0.53-128.19)		36 (63.2)	29 (50.0)	31 (53.4)	
Adenocarcinoma	66 (38.2)	7.31 (0.45-144.01)		17 (29.8)	25 (43.1)	24 (41.4)	
Others	11 (6.4)	3.44 (0.20-38.95)		4 (7.0)	4 (6.9)	3 (5.2)	
**Grade**			**0.031**				0.157
High	84 (48.6)	7.82 (0.90-152.18)		22 (41.5)	29 (55.8)	33 (58.9)	
Low	77 (44.5)	4.05 (0.33-307.61)		31 (58.5)	23 (44.2)	23 (41.1)	
Missing	12 (6.9)						
**Clinical stage**			**<0.001**				**<0.001**
I-II	118 (68.2)	8.03 (0.56-91.76)		30 (52.6)	38 (65.5)	50 (86.2)	
III-IV	55 (31.8)	3.26 (0.21-350.76)		27 (47.4)	20 (34.5)	8 (13.8)	

Bold values were statistically significant (*P* ≤ 0.05).

*SCC* squamous cell carcinoma

### C7 overexpression inhibited proliferation of NSCLC cells *in vitro*

**Figure 4:**
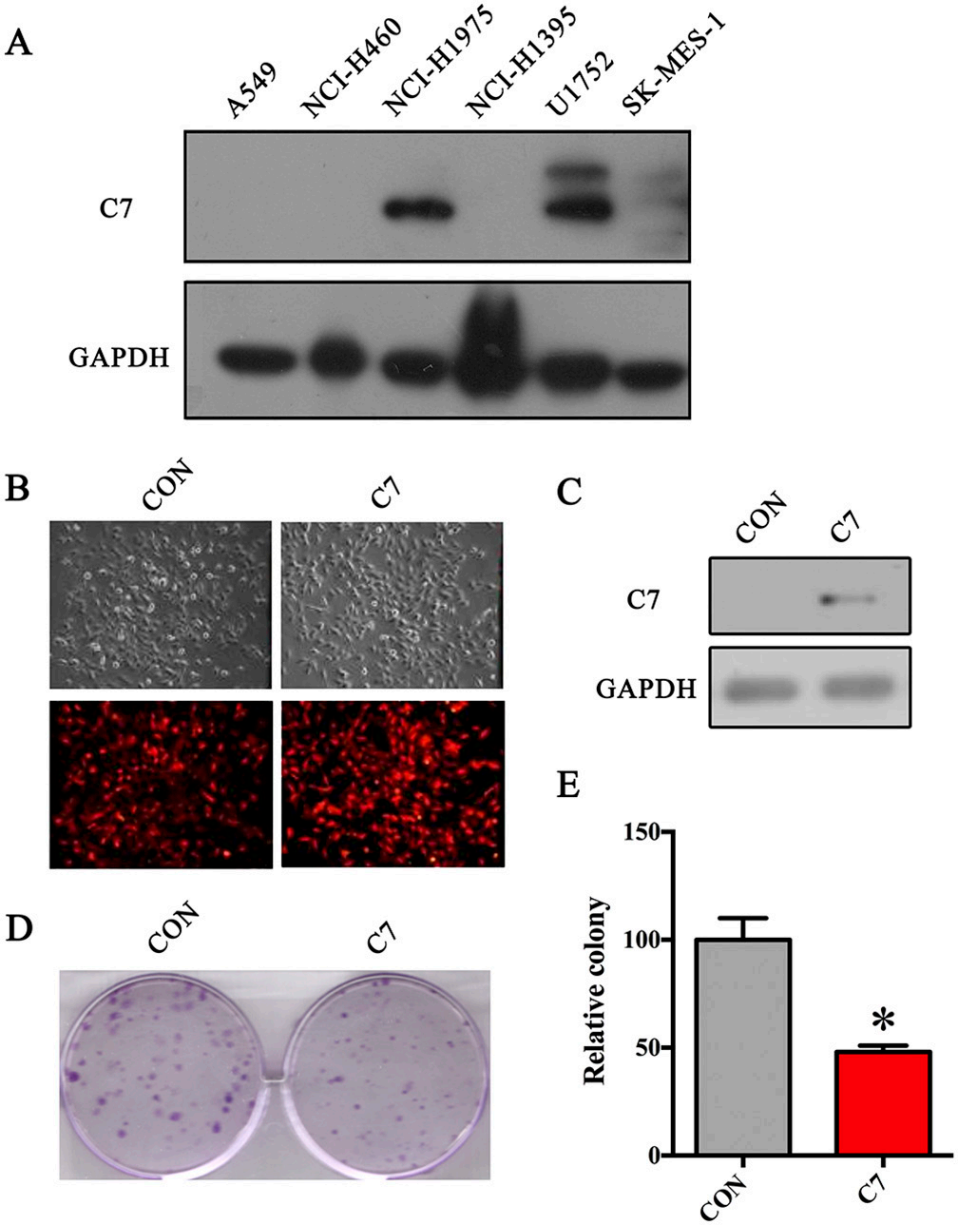
C7 overexpression significantly depressed the proliferation of SK-MES-1 cells **A**. Expression of C7 protein in NSCLC cell lines. **B**. Red fluorescence of SK-MES-1 cells infected by lentiviral vectors. **C**. C7 protein levels examined by western blot. **D**. Colony formation assay of stable SK-MES-1 cells. E. Histogram of colony formation assay. The Student's t-test was used to evaluate the statistical significance. *, *P* < 0.05.

To assess the C7 expression level *in vitro*, a panel of established human NSCLC cell lines was gathered. As Figure 4A indicates, the C7 protein expression is negative in A549, NCI-H460, NCI-H1395 and low in SK-MES-1 compared with that in NCI-H1975 and U1752.

To further evaluate the effect of C7 on the growth of NSCLC cells, the C7-low expressed SK-MES-1 cells were stably transfected by C7 overexpression lentiviral vectors. As shown in Figure 4B, red fluorescence can be observed in almost all stable transfected cells. Furthermore, western bolt showed the objective C7 band in overexpressed SK-MES-1 cells whereas none in control cells (Figure 4C), which confirmed the C7 overexpression in treated cells.

And then, the colony formation assay was done to explore the influence of C7 on proliferation ability of SK-MES-1 cells. As Figures 4D and 4E illustrate, the colonies that the C7 overexpressed SK-MES-1 cells formed were nearly half of that control cells formed, which indicated that the C7 inhibited the growth of NSCLC cells *in vitro*.

## DISCUSSION

Combining the result of FISH and our previous gene-expression profiling data [12], the C7 was identified. In the first place, the clinical and prognostic effects of C7 in ovarian cancer were investigated. As a result, the C7 mRNA content descended gradually in normal, benign, borderline and malignant ovarian tissues. And in patients with ovarian cancer, the decreased expression level of C7 was relative to worse grade. However, it is failing to find any prognostic value of C7 in ovarian cancer from this study and the public database. To further explore the clinical significance of C7 in other malignancies, a number of patients with NSCLC were gathered and consistent downward tendency of C7 mRNA expression was found in the course of lung tumor progression. Apart from tumor grade, clinical stage was also detected correlative to the expression level of C7 in NSCLC patients. And both results from this cohort and the public database demonstrated that NSCLC patients with decreased expression of C7 had a worse outcome. Moreover, the C7 mRNA expression level in cancer tissues was an independent predictor for both disease progression and death in patients with NSCLC. It is speculated from the foregoing that the C7 is a potential tumor suppressor and may make more contributions to NSCLC than ovarian cancer.

Being the central protein of terminal complement cascade, the C7 is an integral part in the formation of MAC and the activation of host defence specifically to the inflammatory region [5, 6, 10]. Besides, the C7 expressed on the cell membrane was reported to have an additional role acting as a regulator of the excessive pro-inflammatory reaction [15]. Therefore, it is concluded that the reduction of C7 expression in tumors prevents the lysis of abnormal cells, enhances the resistance of cancer cells against complement attack, aggravates the inflammation around tumors and finally promotes the malignant progression.

Also, the present study demonstrated that the C7 overexpression suppressed the growth of NSCLC cells *in vitro*. The underlying mechanism hadn't been studied before. It was reported that the sublytic MACs (SC5b-C9) which forms when nucleated cells are not unequivocally identified as non-self [5, 15] can activate a few oncogenic pathways including the mitogen-activated protein kinase (MAPK) family, extracellular regulated protein kinases (ERKs), p38 MAPK, the phosphatidylinositol 3-kinase (PI3K) pathway, Ras [16] and also inhibit apoptosis by causing the phosphorylation of Bad and preventing the activation of Bid, FLIP and caspase-8 [17]. These discoveries provide clues for further understanding of the growth promoting impact of C7.

Identified with this study, Oka et al. found that the C7 expression was noticeably reduced in oesophageal carcinoma with paired normal specimens as control [18]. On the contrary, Suryawanshi et al. observed that the C7 in tumor-related endometriosis had higher expression levels than in normal endometrium [19]. The further reasons underlying the difference need to be explored. Some studies implied that a few complements such as C3a and C5a also contributed to cancer progression through diverse mechanisms including modulating the inflammatory response in the vicinity of tumor and creating favorable microenvironments for tumor development [20–22].

In summary, the present study showed for the first time that the C7 acted as a tumor suppressor, and its expression level was a potential prognostic predictor for both disease progression and death in patients with NSCLC. However, controversy existed that the C7 was identified by integrated genomics while its expression was measured in a RNA level in this study. As a matter of fact, we couldn't find an appropriate kit or approach to detect the copy number variations of C7. In addition, the representativeness of our sample was limited since tissues used in this study were from a single institution. As such, the results remain to be validated, and also, the molecular mechanisms need to further elucidate.

## MATERIALS AND METHODS

### Cell culture

Human ovarian cancer cell line HO-8910 and its derivative HO-8910PM with high metastatic capability were established in our previous studies [23, 24]; human NSCLC cell lines A549, NCI-H460, NCI-H1975, NCI-H1395, U1752 and SK-MES-1 were purchased from the Cell Bank of the Chinese Academy of Sciences, Shanghai, China. SK-MES-1 cells were cultured in Minimum Essential Medium (MEM, Gibco) supplemented with 10% fetal bovine serum (FBS, Gibco); other cells were cultured in RPMI 1640 medium (Gibco) supplemented with 10% FBS. All cells were maintained in a 37°C, 5 % CO_2_ incubator.

### FISH

This assay was performed as previously described [11]. Briefly, chromosomes from HO-8910 and HO-8910PM were prepared with colchicine. Biotin labeled bacterial artificial chromosome (BAC) probes which specifically mapped onto 5p13.1-13.3 (SinoGenoMax Co., Ltd., Beijing, China) were applied to hybridize to the fixed cell nuclei. The signal of hybridization was enlarged through avidin-FITC and observed using a fluorescence microscope (Nikon, Tokyo, Japan) with FITC filter. Images were taken at a magnication of 400 ×. Chromosomes extracted from normal peripheral blood lymphocytes were used as control.

### Tissues and patients

#### Tissues

One hundred and fifty-six malignant ovarian tissues from patients with ovarian cancer, 22 borderline, 33 benign and 24 normal ovarian tissues as well as 173 NSCLC tissues along with corresponding adjacent and normal tissues from patients with NSCLC were applied from the tissue bank of Zhejiang Cancer Hospital. All patients underwent surgery by experienced surgeons of Zhejiang Cancer Hospital and had provided informed consent before operation. This study was approved by the Institutional Review Board of Zhejiang Cancer Hospital.

#### Patients with ovarian cancer

A total of 156 patients with ovarian cancer treated in Zhejiang Cancer Hospital were retrospectively studied. As presented in Table 1, 70.5 % (110/156) were younger than 60 years, 48.7 % (76/156) had residual tumors after surgeries. According to the histological typing of ovarian tumors recommended by World Health Organization (WHO) [25], 73.1 % (114/156) were serous tumor, and 26.9 % (42/156) were non-serous tumor. As for tumor grade, 13.5 % (21/156) were well or moderately differentiated (grade 1-2), 79.5 % (124/156) were poorly differentiated (grade 3), and this information was missing in 11 cases (7.1 %). In accordance with the standard of International Federation of Gynecology and Obstetrics (FIGO) [26], 28 (17.9 %) patients were stage I-II, 128 (82.1 %) patients were stage III-IV.

Follow-up proceeded every three months for the first postoperative year and every six months for the subsequent years until patients died or lost. A routine follow-up evaluation included inquiry, physical examination, blood test, gynecological ultrasoundgraphy. The follow-up data comprised time of follow-up, time of relapse or metastasis, metastatic site, progression free survival (PFS), time of death, causa mortis and overall survival (OS). The median follow-up time was 40 (range 4-75) months. During the follow-up, 51.3 % (80/156) patients progressed, 32.7 % (51/156) died and 9.0 % (14/156) lost.

### Patients with NSCLC

A total of 173 patients with NSCLC who underwent surgical treatment in Zhejiang Cancer Hospital were collected and their clinicopathological characteristics are summarized in Table 3. Among of them, 81.5 % (141/173) were male, 71.7 % (124/173) aged less than 65 years old, 71.1 % (123/173) had smoking experience. On the basis of the WHO classification criteria [27], 55.5 % (96/173) were squamous cell carcinoma, 38.2 % (66/173) were adenocarcinoma, and 6.4 % (11/173) were others. Of the 161 patients with data of tumor grade, 84 patients were high-grade, whereas 77 were low-grade. Based on the TNM classification of IASLC [28], 118 (68.2 %) patients were stage I-II, 55 (31.8 %) patients were stage III-IV.

The median follow-up time of patients with NSCLC was 15 (range 4-30) months. During the follow-up, 23.1 % (40/173) patients progressed, 16.8 % (29/173) died and 1.2 % (2/173) lost.

### Reverse transcriptase quantitative polymerase chain reaction (RT-qPCR)

Total RNA was isolated from tissues using RNeasy Mini kits (QIAGEN Inc., Valencia, CA, USA). Five hundred nanograms of RNA were used for reverse transcription using PrimeScript^TM^ RT Reagent Kit (Takara Biotechnology Co., Ltd., Dalian, China). Quantitative PCR was performed to determine gene expression level on ABI 7500 real-time PCR system (Applied Biosystems Inc., Foster City, CA, USA) using SYBR® Premix Ex Taq^TM^ II (Takara Biotechnology). The sequences of primers were: TGGAAACCCAGTGGCCAGA (C7 forward), GCCATCCATCAGTACAGGTAGAACA (C7 reverse), GAAGGTGAAGGTCGGAGTC (GAPDH forward), GAAGATGGTGATGGGATTTC (GAPDH reverse). The qPCR was carried out under the following conditions: 50°C for 2 minutes, 95°C for 10 minutes, 40 cycles of 95°C for 15 seconds followed by 60°C for 1 minute, and a standard dissociation stage. The results of qPCR were analyzed using the software SDS 2.2.2 (Applied Biosystems) and recorded as threshold cycle (C_T_) values. The expression levels of target gene C7 were adjusted by housekeeping gene GAPDH and compared using the Expression Index (2^-ΔCT^) in accordance with our previous study [11].

### Western blot

A549, NCI-H460, NCI-H1975, NCI-H1395, U1752 and SK-MES-1 cells were harvested by scraping, rinsed three times in phosphate-buffered saline (PBS), and lysed in NP40 cell lysis buffer supplemented with protease inhibitor cocktails. The protein concentration of the cell lysate was measured with the Bradford calorimetric assay (Bio-Rad, Richmond, CA, USA). Thirty micrograms of total protein were electrophoresed on an 8% SDS-polyacrylamide gel electrophoresis (SDS-PAGE) gel, and the separated proteins on the gel were transferred onto a polyvinylidene fluoride membrane (PVDF, Millipore, Bedford, MA, USA). Then the membrane was blocked with TBS-Tween-20 (TBS-T) containing 5% non-fat milk for 2 hours at room temperature, followed by incubation with anti-C7 antibody (Epitomics Inc., Burlingame, CA, USA) overnight at 4°C. Afterwards the membrane was incubated with a HRP-conjugated secondary antibody and detected with enhanced chemiluminescence regent (ECL, Millipore). GAPDH (Sigma, St Louis, MO, USA) was used as a loading control.

### C7 overexpression and colony formation assay

The C7 overexpression lentiviral vector, pLenti-U6-EF1α-C7-FLAG-P2A- tdTomato-T2A-Puromycin, and the empty lentiviral vector for control were purchased from 3D High Throughput Screening Co., Ltd. The lentiviral vectors were co-transfected with helper plasmids Gag pol, VSVG, REV (Invitrogen, Carlsbad, CA, USA) into 293T cells. Then the supernatant was harvested at 48 hours post transfection and purified by centrifugation and filtration using Millex-HV Syringe Filter Units with a 0.45 μm pore size hydrophilic PVDF membrane (Millipore).

SK-MES-1 cells at 40% - 50% confluence were infected overnight with C7 overexpression or control lentiviral in 8 ug/ml polybrene. Stable SK-MES-1 cells were selected with 2 ug/ml puromycin for 2 weeks followed by maintenance in 1 ug/ml puromycin. Then the C7 overexpression in stable SK-MES-1 cells was validated by western bolt.

A total of 2,000 stable SK-MES-1 cells in 2 ml complete medium were seeded onto 6-well plates. After incubating for 2 weeks, cells colonies were fixed with 4 % paraformaldehyde, stained with 0.1 % crystal violet and then colonies containing more than 50 cells were counted. The assay was replicated thrice in triplicate and the Student's t-test was performed to calculate the statistical significance.

### Statistical analysis

Data of C7 mRNA expression from patients’ tissues were analyzed both as continuous and categorical variables. For categorical analysis, the continuous data of C7 expression were divided into low, middle and high groups according to tertile distributions. The difference of C7 expression among tissues with diverse biological behaviors was analyzed using Kruskal-Wallis with Dunn multiple comparisons test. The associations between C7 expression and clinicopathological factors were examined using Mann-Whitney U test and chi-square test. The survival curves based on the C7 expression levels were generated by Kaplan-Meier estimator and the statistical difference was calculated through Log-rank test. A Cox proportional hazards regression model was performed to acquire the prognostic value of the C7 expression. The public database and web application named KM plotter (http://kmplot.com/analysis/) was used to verify the prognostic results [29]. SPSS 21.0 for Windows (IBM) was used for data analyses, and a *P* value ≤ 0.05 in a two-tailed test was deemed of statistical significance.
